# Evaluating the effects of short-term low temperature on the growth and development of *Trichopria drosophilae* based on the age–stage two-sex life table

**DOI:** 10.1186/s13071-024-06480-6

**Published:** 2024-10-05

**Authors:** Qiang Chen, Jinlong Zhang, Ye Tian, Juan Li, Weizhi Ning, Guohua Chen, Xiaoming Zhang

**Affiliations:** 1 College of Plant Protection, State Key Laboratory of Yunnan Biological Resources Protection and Utilization, Yunnan Agricultural University, Kunming, 650201 China; 2 Tea Research Institute, Yunnan Academy of Agricultural Sciences, Kunming, 650205 China

**Keywords:** Age-stage two-sex life table, Global warming, Short-term low temperature, *Trichopria drosophilae*

## Abstract

**Background:**

The effects of low temperatures on parasitic wasps are crucial for maintaining farmland biodiversity and enhancing biological control, especially given the implications of global warming and frequent extreme cold events.

**Methods:**

We studied the effects of different low temperatures (−8 ± 1 °C, −4 ± 1 °C, 0 ± 1 °C, 4 ± 1 °C, and 8 ± 1 °C) on the mating frequency and duration of male adults of *Trichopria drosophilae* and the number of pupae beaten by female adults, and constructed the age–stage two-sex life table of *T. drosophilae*.

**Results:**

This study found that male *T. drosophilae* adults exposed to low temperatures for 12 h significantly altered their mating behavior, peaking between 15:00 and 17:00. As the temperature dropped during the exposure, both the mating frequency of *T. drosophilae* and the duration of pupal beating were affected. The survival rate of female adults dropped from 39.55% at 8 °C to just 21.17% at −8 °C. Low-temperature treatment shortened the development period and lifespan for *T. drosophilae* adults. They developed 4.71 days faster and had a total lifespan that was 10.66 days shorter than those in the control group after being exposed to −8 °C. Furthermore, the average number of eggs laid by females at −8 °C was 4.46 less than that at 8 °C and 6.16 less than that in the control group, which laid an average of 21.55 eggs. The net reproductive rate (*R*_0_) of *T. drosophilae* decreased with lower temperatures, reaching a low of 23.64 at −8 °C. Conversely, the intrinsic growth rate (*r*_m_) actually increased as temperatures dropped, with the lowest value being 0.21 at −8 °C.

**Conclusions:**

The findings indicate that short-term exposure to low temperatures hampers the growth and population increase of *T. drosophilae*, thereby reducing their effectiveness as biological control agents.

**Graphical Abstract:**

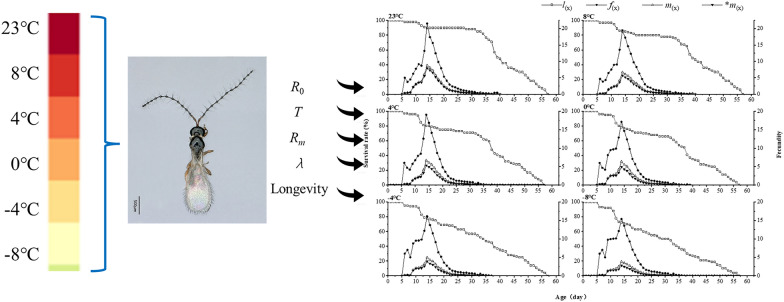

## Highlights

1. We studied how short-term exposure to low temperatures affects the mating behavior of the parasite *Trichopria drosophilae* and found that it disrupts their mating process.
2. Using data on age and developmental stage, we developed a two-sex life table for *T. drosophilae* under short-term low temperatures.
3. Our findings indicate that short-term low temperatures reduce the population growth of *T. drosophilae*.

## Background

The global fruit industry faces a serious threat from *Drosophila suzukii* (spotted wing drosophila, SWD). This pest is causing significant losses, particularly as China expands its soft fruit cultivation. In Yunnan Province’s Shiping County [1, 2], a survey found that *Morella rubra* fresh fruit had a 100% damage rate during a peak outbreak of *D. suzukii* [3]. In 2016, this pest caused economic losses of 8.92 billion yuan to China’s *Prunus pseudocerasus* [4]. In the United States, *D. suzukii* was first reported in California in 2008, resulting in a 30% loss for the local *P. pseudocerasus* industry [5]. In Europe, the European Union has listed *D. suzukii* as an A2 quarantine pest due to its significant spread and impact [6].

The parasitic wasp *Trichopria drosophilae* (Hymenoptera: Diapriinae) can find and parasitize *D. suzukii* pupae, even when hidden inside fruit or buried in the soil [7]. After immobilizing the host, the wasp lays its eggs inside the pupa. *Trichopria drosophilae* is one of the most important natural enemies of *D. suzukii* and is found in many countries, including China, South Korea, Israel, France, Germany, Greece, Italy, the Netherlands, Spain, Morocco, Mexico, and the United States [8, 9]. Recent studies have shown that *T. drosophilae* can effectively control *D. suzukii* populations. For instance, after releasing large numbers of *T. drosophilae* in *P. pseudocerasus* orchards in southern Italy, the rate of fruit pest damage decreased by 34% [10]. Research by Wang revealed that *T. drosophilae* is more efficient than another wasp, *Pachycrepoideus vindemmiae*, in locating and parasitizing host pupae [11]. The parasitic efficiency of *T. drosophilae* increases with temperatures between 15 °C and 30 °C, which shortens both its pre-parasitic period and lifespan. However, at 35 °C, the survival rate of *T. drosophilae* drops to 0%, indicating that this temperature exceeds its tolerance limits. The optimal temperature for the growth and reproduction of *T. drosophilae* is around 23 °C [12].

Parasitic wasps are sensitive to temperature, making it difficult for them to maintain a stable body temperature. Short-term exposure to low temperatures in their natural habitat can disrupt their reproductive behavior [13]. When the temperature drops, adult insects become less active, and their sexual organs and gonads do not fully develop. This can prevent male and female adults from mating, or if they do mate, the rates of hatching and egg-laying are reduced [14]. Hao found that when *Drosophila melanogaster* were exposed to 5 °C for 24 h, they became inactive and could not communicate with one another [15]. This lack of activity led to reduced mating behavior between male and female *D. melanogaster* for up to 72 h after the temperature drop.

The life table, introduced by Pearl and Parker in 1921, is an important tool for studying the growth and reproduction of insect populations [16]. However, traditional life tables often overlook factors like developmental rates and male populations, which can lead to significant errors in calculations. The age–stage two-sex life table offers a more accurate method by considering age differences and sex ratios in population growth, as well as generation overlap [17]. Recently, many researchers have used this method to analyze the growth, development, and reproductive capacity of various insects, including *Frankliniella intonsa* [18], *Riptortus pedestris* [19], *Aedes aegypti* [20], and *Trichogramma achaeae* [21]. In the case of *T. drosophilae*, Fang is the only researcher to have investigated its life table under constant laboratory temperatures, ignoring how short-term low temperatures affect the species’ population dynamics and parameters of the age–stage two-sex life table [12]. Since short-term low temperatures can significantly impact reproduction, further research is needed to understand their effects on the growth and development of *T. drosophilae*. This research will provide a scientific foundation for mass breeding, low-temperature storage, and efficient use, and will help avoid unsuitable short-term low-temperature conditions during field releases.

## Methods

### Insect cultures

In the summer of 2016, adult colonies of *D. suzukii* were collected from *Morella rubra* fruit in Dianwei village, Kunming, Yunnan province, Southwest China (coordinates: 102° 9′ E, 24° 8′ N). The adult *T. drosophilae* colony was also obtained from infested fruit collected at the same location. The *D. suzukii* specimens were maintained in incubators set at a temperature of 23 ± 1 °C, with relative humidity (RH) of 70 ± 5%, and a light cycle of 16 h of light followed by 8 h of darkness (L:D). The colonies were raised on a maize flour-based artificial diet and housed in 50-ml plastic centrifuge tubes (12 cm height, 3 cm diameter). This diet included 1000 ml of water, 15 g of yeast per liter, 21 g of agar per liter, 90 g of sucrose per liter, 180 g of semolina flour per liter, and 15 g of raisins per liter. Flies were introduced into these tubes twice a week; the tubes were sealed with fine mesh and left for 2–3 days to encourage egg-laying. *Trichopria drosophilae* colonies were reared using *D. suzukii* as their host, feeding on a diet infested with *D. suzukii* larvae and pupae. Adult parasitoids were removed 2 weeks prior to the emergence of the new generation. Adult *T. drosophilae* received a solution of 10% honey on a cotton ball for nourishment [22]. The temperature and RH inside all climate cabinets were monitored every 30 min using a COS-03 temperature/RH system (Shandong Renke Control Technology Co., Ltd., China).

### Effects of short-term low temperature on the mating frequency and duration of male adults of *T. drosophilae*

Fang’s study [12] examined the effects of various low-temperature environments on the mating behavior of *T. drosophilae*. The temperatures tested were −8 ± 1 °C, −4 ± 1 °C, 0 ± 1 °C, 4 ± 1 °C, and 8 ± 1 °C. Male adult *T. drosophilae* were first exposed to these temperatures for 12 h in an artificial climate incubator. After this exposure, the males were paired with females in a 10:1 ratio and placed in an insect rearing tank. The researchers recorded the mating frequency of the male adults during three time periods: morning (9:00–11:00), afternoon (15:00–17:00), and evening (20:00–22:00). To aid in observations, a cold light source was used, and the visual field was adjusted to 2.5 × 20 times under a stereoscopic microscope. A fixed camera was used to track and capture mating behavior. Each observation session included 10 pairs of insects, and this was carried out three times. Additionally, the number and duration of mating at 23 °C served as the control for comparison [23].

### Effects of short-term low temperature on the duration of beating pupae of *T. drosophilae* female adults

Female *T. drosophilae* were exposed to various low-temperature environments for 12 h. Male and female *T. drosophilae* were raised separately. During the peak mating period, from 15:00 to 17:00 PM, equal numbers of male and female *T. drosophilae* were paired and placed in a rearing tank with *D. suzukii* pupae. After mating, the parasitic behavior of the female *T. drosophilae* was observed using an anatomical microscope, and a fixed camera recorded the duration of the pupal stage. Each treatment consisted of 10 pairs and was replicated three times. The duration of pupal development at 23 °C served as a control [24].

### Effect of short-term low temperature on age–stage two-sex life table of *T. drosophilae*

The experiment was conducted in a controlled environment with humidity levels of 60% ± 5%, 16 h of light and 8 h of darkness each day, and a light intensity of 18,000 lx. The temperature was maintained at 23 °C. A total of 100 pupae of *D. suzukii*, infested with *T. drosophilae*, were collected at 23 °C and placed in 750 ml glass bottles. These bottles were then positioned in incubators set to various low temperatures (−8 ± 1 °C, −4 ± 1 °C, 0 ± 1 °C, 4 ± 1 °C, and 8 ± 1 °C). Adult emergence was observed daily at 8:00 and 20:00. The emerging male and female adults were paired in a 1:1 ratio and placed in a rearing cage. New *D. suzukii* pupae were added daily to allow newly emerged *T. drosophilae* to infest them, and the parasitoids were provided with a 10% honey-water solution for nutrition. When the *D. suzukii* pupae turned black, it indicated that the parasitoid had entered the pupal stage. The duration from larval pupation to eclosion was measured as the pupal stage. Data on parasitism and survival were collected until all adults had died [25].

### Life table analysis

The TWOSEX-MSChart program was used to create an age–stage two-sex life table [26], following the methods outlined by Chi and Liu [27] and Chi [28]. This life table includes several important population parameters: age–stage-specific survival rate (*s*_*xj*_, *x* = age and *j* = stage), age-specific survival rate (*l*_*x*_), age–stage-specific fecundity (*f*_*xj*_), age-specific fecundity (*m*_*x*_), age–stage life expectancy (*e*_*xj*_), and reproductive value (*v*_*xj*_). Additionally, the life table parameters include net reproductive rate (*R*_0_), intrinsic rate of increase (*r*), finite rate of increase (*λ*), and mean generation time (*T*).

To calculate the values for *m*_*x*_ and *l*_*x*_ in the age–stage two-sex life table [27], the following calculations were performed:

$${m}_{x}=\frac{ {\sum }_{j= 1}^{k}{s}_{xj} {f}_{xj}}{{\sum }_{j= 1}^{k}{s}_{xj} } {l}_{x}={\sum }_{j= 1}^{k}{s}_{xj},$$

where the variable *k* represents the number of stages, *s*_*xj*_ denotes the probability of survival and growth from birth to age *x* and stage* j*, and* f*_*xj*_ represents the average number of offspring produced by a female at age *x*. Subsequently, the net reproductive rate (*R*_0_) was computed using the following equation:

$${R}_{0}={\sum }_{x= 0}^{\infty }{l}_{x}{m}_{x}.$$

The intrinsic rate of increase (*r*) was estimated by applying the Euler–Lotka equation, where the age is indexed from 0 [29]:

$${\sum }_{x= 0}^{\infty }{e}^{-r(x+1)}{l}_{x}{m}_{x}= 1.$$

The finite rate of increase (*λ*) was computed using the equation *λ* = *e*^*r*^. The mean generation time (*T*) was defined as the duration required for a population to increase its size by *R*_0_ times (*e*^*rT*^ = *R*_0_ or *λ*^*T*^ = *R*_0_) while maintaining a stable age–stage distribution. The formula utilized to calculate the value of *T* was as follows:

$$T=\frac{\text{ ln}{R}_{0} }{r},$$

where *e*_*xj*_ denotes the duration for which an individual of age *x* and stage *j* is expected to live. The value of *e*_*xj*_ was determined using the formula described by Chi and Su [30]:

$${e}_{xj}={\sum }_{i=x}^{\infty }{\sum }_{y=j}^{k}{ s{\prime}}_{iy},$$

where *s*′_*iy*_ is the probability that an individual of age *x* and stage *j* will survive to age *i* and stage *y*, assuming that *s*′_*xj*_ = 1 [30]. The reproductive value (*v*_*xj*_) is defined as the contribution of individuals at age *x* and stage *j* to the future population, and its calculation formula, as provided by Tuan [31], is presented below:

$${v}_{xj}=\frac{{e}^{r(x+1)}}{{s}_{xj}}{\sum }_{i=x}^{\infty }{e}^{-r(i+1)}{\sum }_{y=j}^{k}{{s{\prime}}_{iy}f}_{iy}.$$

### Data analysis

The statistical analysis was performed using SPSS 25.0 software to calculate the mean and standard error (SE) of all data points. We used analysis of variance to examine the frequency of mating, mating duration, and pupal duration of *T. drosophilae* at different temperatures. The least significant difference (LSD) method was employed to determine the significance of any differences observed. For analyzing the age–stage two-sex life table data, as well as the developmental duration, total longevity of eggs, larvae, pupae, and adults, net reproductive rate, mean generation time, intrinsic growth rate, and finite growth rate, we utilized TWOSEX-Mschart software. To assess significance, we conducted a bootstrap paired test with 100,000 iterations (*P* < 0.05) using a bootstrap program. Finally, Origin 2018 software was used for creating graphs and visual representations of the data [32, 33].

## Results

### Effects of short-term low-temperature treatment on the mating frequency and duration of male adults of *T. drosophilae*

**Fig. 1:**
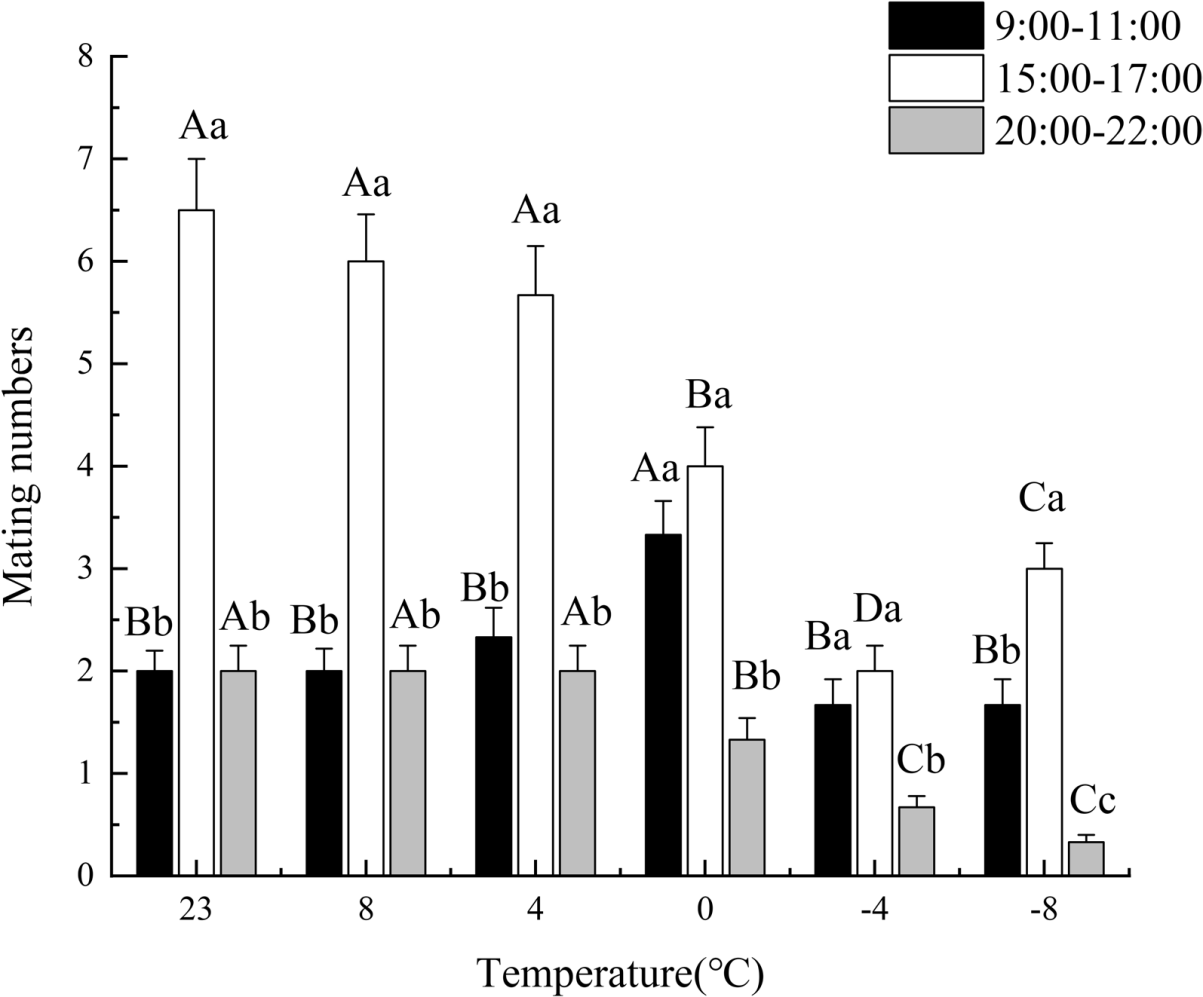
Effects of short-term low temperature on mating frequency of male adults of *T. drosophilae*. The data in the figure are mean + SE bars. In the figure, different uppercase letters indicate significant differences in the mating frequency of *T. drosophilae* treated with different short-term low temperatures during the same period using the LSD method (*P* < 0.05). On the other hand, lowercase letters denote significant differences in the mating frequency of *T. drosophilae* at the same temperature and different periods, also after applying the LSD method (*P* < 0.05)

Short-term low temperatures exposure significantly affected the mating frequency of adult male *T. drosophilae* (Fig. 1). The mating frequency decreased as temperatures fell from 8 °C to −8 °C. The peak mating activity occurred between 15:00 and 17:00, with the highest mating frequency of 6.00 observed at 8 °C during this time. Mating frequencies at 8 °C and 4 °C together accounted for over 50% of the total mating activity. At 0 °C, −4 °C, and −8 °C, mating frequency between 20:00 and 22:00 was significantly lower than during the other two time periods (0 °C: *F*_(2,6)_ = 21.15, *P* = 0.0021; −4 °C: *F*_(2,6)_ = 20.55, *P* = 0.0019; −8: *F*_(2,6)_ = 57.30, *P* = 0.0001). Furthermore, from 9:00 to 11:00, the mating frequency at 0 °C was significantly higher than at other temperatures and control groups (*F*_(2,4)_ = 6.00, *P* = 0.0256). During the period from 15:00 to 17:00, the mating frequency of insects at 8 °C and 4 °C and the control group was significantly greater than that of insects kept at other temperatures (*F*_(4,10)_ = 131.50, *P* = 0.0001), showing 5.67-fold greater frequency. In contrast, between 20:00 and 22:00, the mating frequency of insects at −8 °C was significantly lower than at other temperatures (*F*_(4,10)_ = 8.67, *P* = 0.0027), with frequency that was 0.33 times lower.

Brief low-temperature exposure affected the mating duration of *T. drosophilae*. As illustrated in Table 1, the time spent mating increased as temperatures fell between 4 °C and −8 °C. At 8 °C, there were no significant differences in mating duration during the time intervals of 9:00–11:00, 15:00–17:00, and 20:00–22:00 (*F*_(2,6)_ = 0.52, *P* = 0.6191). Similarly, at 4 °C, the duration of mating remained consistent across the same time intervals (*F*_(2,6)_ = 3.06, *P* = 0.1212). However, at 0 °C, the mating duration from 9:00 to 11:00 was significantly shorter than from 20:00 to 22:00 (*F*_(2,6)_ = 11.63, *P* = 0.0086). At −4 °C and −8 °C, there were no significant differences in mating duration among the time intervals (*F*_(2,6)_ = 3.25, *P* = 0.1106 and *F*_(2,6)_ = 3.00, *P* = 0.1250, respectively). The longest mating duration was recorded at −8 °C, with the durations during the intervals 15:00–17:00 and 20:00–22:00 being significantly longer than the control (15:00–17:00: *F*_(5,12)_ = 12.13, *P* = 0.0002; 20:00–22:00: *F*_(5,12)_ = 8.32, *P* = 0.0013). Furthermore, the mating duration from 9:00 to 11:00 was significantly longer than that at 4 °C (9:00–11:00: *F*_(5,12)_ = 8.10, *P* = 0.0015).

**Table 1:** Effects of short-term low temperature on the duration of mating behavior of *T. drosophilae*

Time interval	Temperature (°C)					
	23	8	4	0	−4	−8
9:00–11:00	3.00 ± 0.29 ABa	3.00 ± 0.58 ABa	2.33 ± 0.33 Ba	3.33 ± 0.33 ABb	4.33 ± 0.33 Aba	5.00 ± 0.00 Aa
15:00–17:00	3.17 ± 0.33 Ba	3.67 ± 0.33 Ba	3.50 ± 0.29 Ba	4.50 ± 0.29 ABab	5.00 ± 0.00 Aa	5.50 ± 0.29 Aa
20:00–22:00	3.17 ± 0.17 Ba	3.50 ± 0.50 ABa	3.50 ± 0.50 ABa	5.17 ± 0.17 Aa	4.50 ± 0.00 ABa	5.50 ± 0.00 Aa

The data in the table represent the mean duration of the mating behavior of *T. drosophilae*, with standard errors included. The duration of the mating behavior was measured in minutes. Lowercase letters are used to indicate significant differences in duration at the same temperature, while uppercase letters represent significant differences in duration at the same time, based on the LSD method (*P* < *0.05*). This analysis was conducted under short-term low-temperature stress at the same temperature and period

### Effects of short-term low-temperature treatment on the duration of beating pupae of *T. drosophilae*

**Fig. 2:**
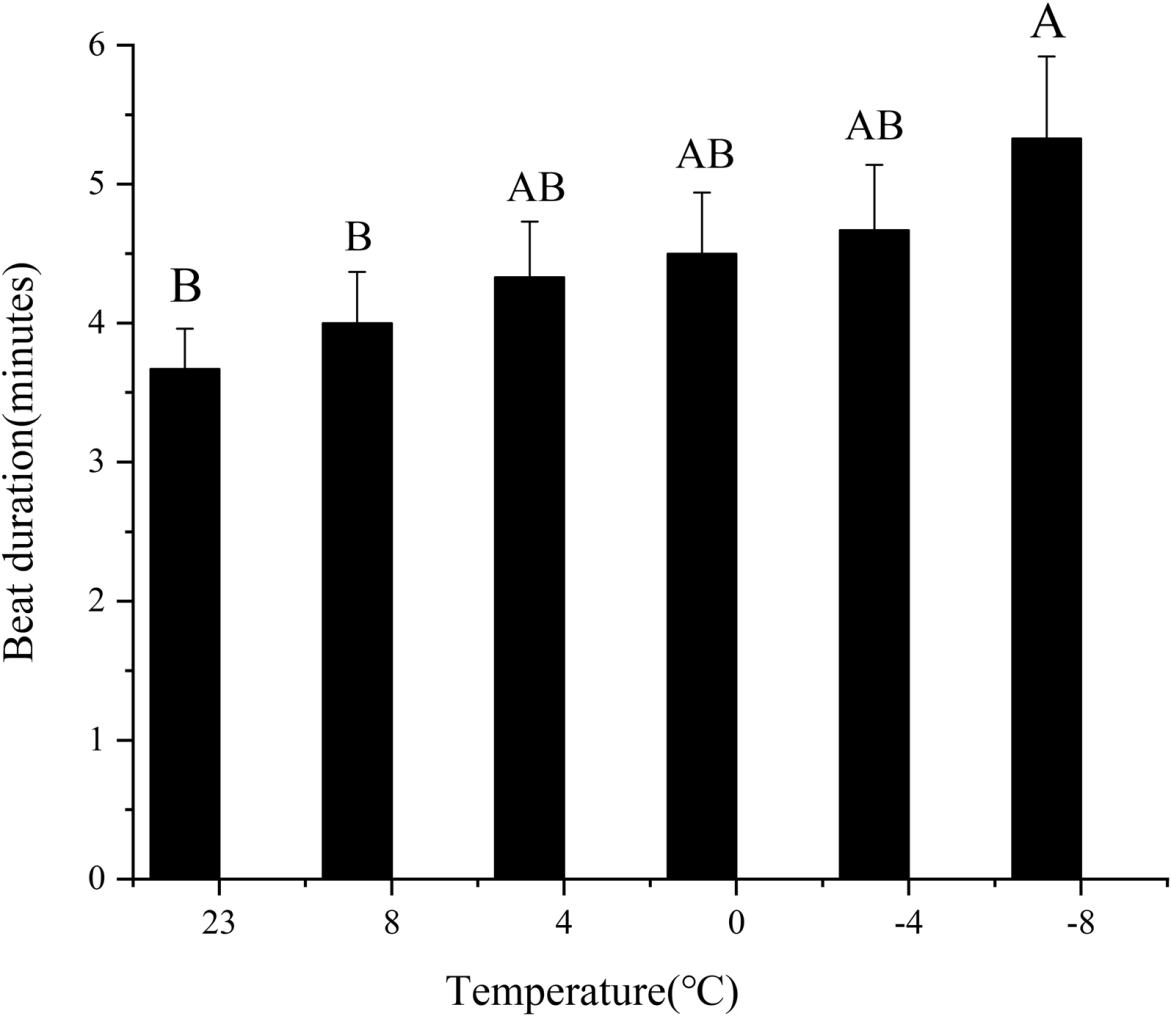
Effect of short-term low temperature on the duration of beating pupae of *T. drosophilae*. The data in the figure are mean + SE. The uppercase letters in the picture indicate significant differences in the duration of pupation of *T. drosophilae* under various short-term low-temperature stress during the same peak mating period of 15:00–17:00. This was determined after using the LSD method (*P* < 0.05)

Short-term low-temperature treatment affected the flapping time of *T. drosophilae* beating pupae (Fig. 2). The study found that as the temperature dropped from 8 °C to −8 °C, the flapping time of the pupae increased. At −8 °C, the longest recorded flapping time was 5.33 min. In contrast, at 23 °C and 8 °C, the shortest flapping times were 3.67 min and 4.00 min, respectively. Additionally, the study showed that the flapping time of *T. drosophilae* treated at −8 °C was significantly longer than that of the control group (*F*_(5,12)_ = 1.79, *P* < 0.05).

### Effects of short-term low-temperature treatment on developmental duration and longevity of *T. drosophilae* F_1_

Short-term low-temperature treatment was found to affect the developmental stages of *T. drosophilae*. At 23 °C, the average development duration for the egg and larval stages was 5.16 ± 0.17 days, the pupal stage lasted 9.69 ± 0.52 days, and the adult stage was 28.62 ± 0.49 days. After treatment at 8 °C, the egg-larval development period increased to 5.67 ± 0.16 days, the pupal stage extended to 10.56 ± 0.52 days, and the adult stage averaged 28.23 ± 0.59 days. Following treatment at 4 °C, the egg-larval period increased to 5.70 ± 0.19 days, the pupal stage lasted 10.36 ± 0.54 days, and the adult stage duration was 27.79 ± 0.66 days. At 0 °C, the egg-larval development took 5.84 ± 0.22 days, the pupal duration was 11.13 ± 0.68 days, and the adult stage lasted 27.23 ± 0.89 days. Treatment at −4 °C led to an egg-larval development time of 6.27 ± 0.25 days, a pupal duration of 11.58 ± 0.69 days, and an adult stage duration of 24.38 ± 0.78 days. After exposure to −8 °C, the egg-larval development period increased to 6.73 ± 0.31 days, the pupal stage lasted 13.09 ± 0.92 days, while the adult stage time was 23.91 ± 0.93 days. At this temperature, the egg-larval development was 1.57 days longer than the control group, the pupal duration was 3.40 days longer, and the adult stage was 4.71 days shorter. Additionally, low-temperature treatment influenced the overall lifespan of *T. drosophilae*; at 23 °C, the average lifespan was 40.15 days, significantly longer than that at lower temperatures (*P* < 0.05) (Table 2).

**Table 2:** Developmental duration of F_1_ generation of *T. drosophilae* under short-term low-temperature stress at each developmental stage

Temperature (°C)	Developmental duration (days)			Total longevity
	Egg–larvae	Pupa	Adult	
23	5.16 ± 0.17 cd	9.69 ± 0.52 a	28.62 ± 0.49 a	40.15 ± 1.23 a
8	5.67 ± 0.16 bc	10.56 ± 0.52 a	28.23 ± 0.59 ab	37.89 ± 1.46 b
4	5.70 ± 0.19 bc	10.36 ± 0.54 a	27.79 ± 0.66 ab	35.78 ± 1.56 b
0	5.84 ± 0.22 ab	11.13 ± 0.68 a	27.23 ± 0.89 ab	33.71 ± 1.82 bc
−4	6.27 ± 0.25 ab	11.58 ± 0.69 a	24.38 ± 0.78 bc	32.58 ± 1.52 c
−8	6.73 ± 0.31 a	13.09 ± 0.92 a	23.91 ± 0.93 c	29.49 ± 1.57 cd

The data in the table are the mean ± SE of the developmental duration of the F_1_ generation of *T. drosophilae*, and the different letters in the same column indicate that there are significant differences at *P* < 0.05 level by paired bootstrap test

**Fig. 3:**
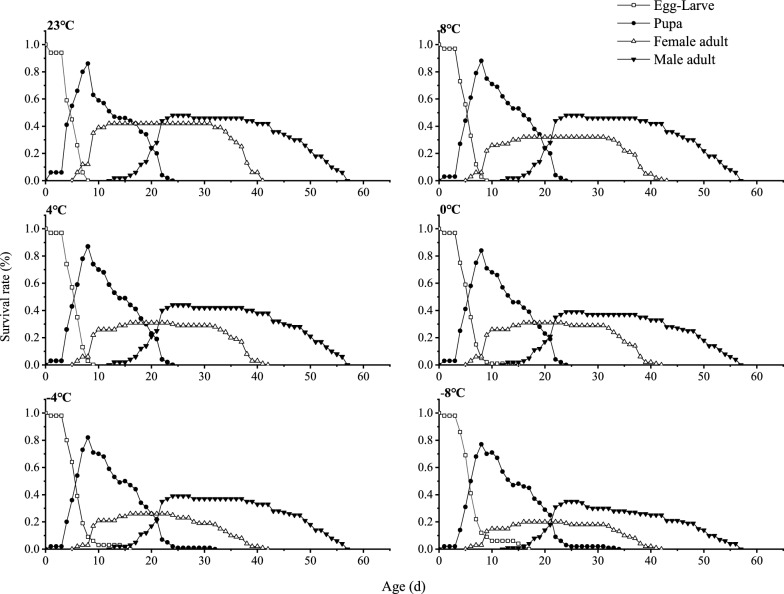
Effects of short-term low temperature on the survival rate (*S*_*xj*_) of *T. drosophilae*

The growth curves of *T. drosophilae* under different temperature conditions show significant overlaps, highlighting the complex and variable relationships between individual growth patterns. Except for the egg-larval stage, the specific age–stage survival rate (*S*_*xj*_) for developmental stages generally increases before decreasing with the progression of development time. As temperatures range from 8 °C to −8 °C, the survival rate of *T. drosophilae* decreases as the temperature decreases. For female adults, the survival rate declines from 39.55% at 8 °C to 21.17% at −8 °C (Fig. 3).

**Fig. 4:**
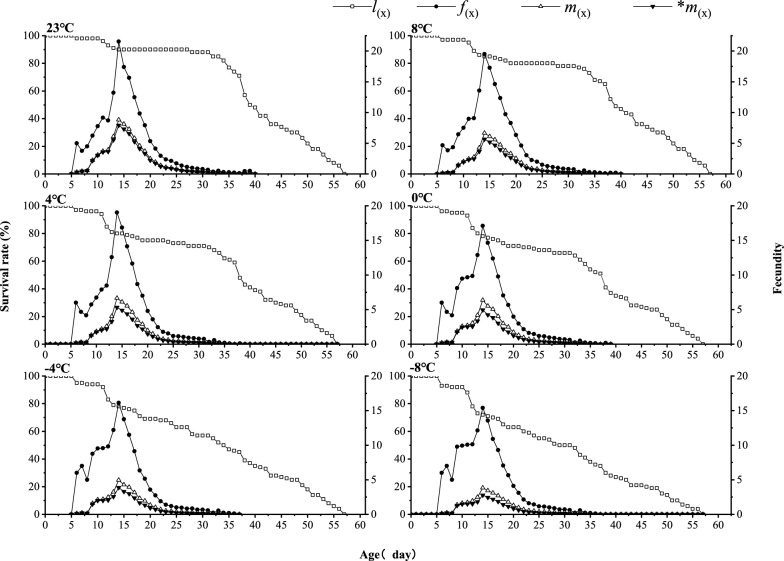
Effects of short-term low temperature on the age-specific survival rate (*l*_*x*_), age-specific fecundity (*f*_*x*_), and age-specific fecundity (*m*_*x*_) of F_1_ generation of *T. drosophilae*

After brief exposure to low temperatures, the specific age–stage fecundity (*f*_*x*_) and specific age fecundity (*m*_*x*_) of *T. drosophilae* females first increase and then decrease. The peak age-specific fecundity (*f*_*x*_) for females at 8 °C occurs at 15 days, reaching 19.85 eggs. At −8 °C, the maximum *m*_*x*_ for females is observed at 14 days, with 15.39 eggs. The age-specific fecundity (*f*_*x*_) of females at −8 °C is 4.46 eggs less than that of females treated at 8 °C, and 6.16 eggs less than the peak of females at 8 °C (21.55 eggs) (Fig. 4).

**Fig. 5:**
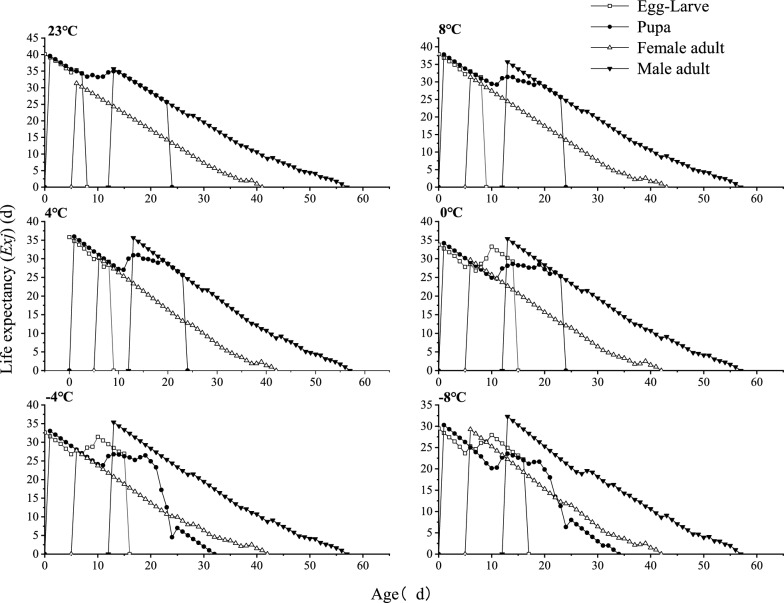
Effects of short-term low-temperature treatment on the specific age–stage life expectancy (*e*_*xj*_) of* T. drosophilae*

The specific age–stage life expectancy (*e*_*xj*_) of *T. drosophilae* under short-term low and high temperatures indicates a gradual decline in life expectancy during the egg-larval and pupal stages, followed by an increase in the middle stages. Male adults exhibit a significantly higher life expectancy than female adults, and life expectancy for both sexes decreases over time during each temperature treatment along the growth time axis (Fig. 5).

### Effects of short-term low-temperature treatment on life table parameters of *T. drosophilae* population

Short-term exposure to low temperatures had a significant impact on the life table parameters of *T. drosophilae* (Table 3). The net *R*_0_ value decreased as the temperature dropped, with the highest recorded value of 46.37 ± 7.11 at 8 °C and the lowest of 23.64 ± 5.40 at −8 °C, both of which were lower than the control value of 64.75 ± 7.83. Similarly, the average generation period (*T*) also decreased with lower temperatures; the average period was longest at 15.11 ± 0.40 at 8 °C and shortest at 13.62 ± 0.54 at −8 °C. However, these comparisons to the control value of 14.60 ± 0.34 were not statistically significant *(P* > 0.05). In contrast, the intrinsic rate of increase (*r*_m_) rose with lower temperatures, with a value of 0.21 ± 0.02 at −8 °C, which was significantly lower than the control rate of 0.28 ± 0.011 (*P* < 0.05). Additionally, the finite growth rate (λ) decreased at lower temperatures, reaching minimum values of 1.26 ± 0.02 at −4 °C and 1.24 ± 0.02 at −8 °C, both significantly below the control value of 1.33 ± 0.01 (*P* < 0.05). In summary, short-term exposure to low temperatures suppressed the population growth of *T. drosophilae*.

**Table 3:** Effects of short-term low temperature on life table parameters of *T. drosophilae* population

Temperature (°C)	Net reproductive rate* R*_0_	Mean generation time *T*	Intrinsic increase rate *r*_m_	Finite increase rate *λ*
23	64.75 ± 7.83 a	14.60 ± 0.34 a	0.28 ± 0.011 a	1.33 ± 0.01 a
8	46.37 ± 7.11 ab	15.11 ± 0.40 a	0.25 ± 0.01 ab	1.29 ± 0.02 ab
4	40.98 ± 6.78 c	14.98 ± 0.35 a	0.24 ± 0.01 b	1.28 ± 0.02 b
0	39.26 ± 6.43 c	14.27 ± 0.38 a	0.25 ± 0.01 ab	1.29 ± 0.02 ab
−4	30.27 ± 5.84 cd	14.48 ± 0.41 a	0.23 ± 0.01 b	1.26 ± 0.02 b
−8	23.64 ± 5.40 d	13.62 ± 0.54 a	0.21 ± 0.02 b	1.24 ± 0.02 b

The data in the table are mean ± SE. Different letters in the same column indicate that there are significant differences at the *P* < 0.05 level by paired bootstrap test

## Discussion

Short-term low temperatures affect the mating behavior of *T. drosophilae* at different life stages. The main mating activity occurs between 15:00 and 18:00, similar to other parasitic wasps such as *Tetrastichus hagenowii* [34] and *Coccophagus japonicus* [35]. This peak may be linked to the production of female sex hormones. Different insect species exhibit varied mating behaviors in response to environmental temperatures. For instance, *Callosobruchus chinensis* [36] has a shorter mating duration at lower temperatures, which aligns with our observations of *T. drosophilae*. Similarly, *Bactrocera tryoni* [37] shows a decrease in mating frequency at lower temperatures, and we noted a similar decline in *T. drosophilae* mating frequency when temperatures ranged from 8 °C to −8 °C. This reduction may result from the inhibitory effects of short-term low temperatures on the release of female hormones in *T. drosophilae* and a decrease in male vitality.

Once *T. drosophilae* identifies a potential host, it assesses the host's health, developmental stage, and size to determine whether it is suitable for parasitism. The wasp first taps the host pupae, an important behavior that helps prevent unsuitable parasitism and ensures the proper development of its offspring [38]. It uses its antennae, keen eyesight, and ovipositor receptors to evaluate the host's suitability before deciding whether to lay eggs. If the host is found to be unsuitable, the wasp quickly abandons it in search of a more appropriate host. Recent studies indicate that brief exposure to low temperatures can extend the duration of *T. drosophilae*’s pupae-tapping behavior and reduce its parasitic effectiveness. Research by Shen et al. [39] showed that stress can significantly lower the host selection rate of *Trichogramma chilonis* adults and prolong the time it takes for them to find and court potential mates. These effects are likely due to reduced energy expenditure and the need to maintain physiological balance.

Studying insect life tables is crucial for understanding how insects develop, survive, and reproduce in different environments. This research is also important for examining the relationships between different species and the dynamics of insect populations [40]. In this study, we focused on *T. drosophilae* after exposure to various short-term low-temperature treatments. We created life tables that displayed the age–stage survival rate (*S*_*xj*_) and age-specific survival rate (*l*_*x*_) to show the complete life cycle of *T. drosophilae*, from egg to adult, under different temperatures. Our investigation highlights how short-term low temperatures affect the growth, development, and survival of *T. drosophilae*. Although *T. drosophilae* can complete its life cycle and reproduce, low temperatures significantly impact factors like development time, survival rate, and reproductive output. Changes in temperature stress result in differences in the growth and reproductive capabilities of the species. Specifically, exposure to low temperatures extends the egg-larval and pupal stages but reduces the adult stage and overall lifespan of *T. drosophilae*. Other parasitoids, such as *Fopius vandenboschi* [41], also show reduced adult longevity under low temperatures. This decline may occur because *T. drosophilae* must use significant lipid reserves and energy to survive brief periods of cold, which adults cannot replenish since they do not produce lipids internally. This leads to a trade-off in resource allocation between survival and reproduction, ultimately shortening their lifespan [42]. Generally, a longer lifespan in adult parasitic wasps improves their effectiveness in controlling target pest populations. Their ability to manage host populations relies heavily on their feeding, parasitism, and predatory abilities during the adult stage [43]. Moreover, a longer adult lifespan supports successful establishment of insect populations in outdoor settings. Therefore, short-term low temperatures reduce the adult lifespan of *T. drosophilae*, which diminishes its effectiveness as a biological control agent.

The survival rate is a common metric used to evaluate how resilient natural enemy insects like *T. drosophilae* are to temperature changes. It reflects their sensitivity to temperature fluctuations and is vital for understanding their survival prospects [44, 45]. When temperatures rise above a certain critical level, the survival of these insects is at risk, which is essential for the growth of their populations. This study found that short-term exposure to low temperatures can decrease the survival rate of *T. drosophilae*, aligning with previous research by Sun [40]. This indicates that lower temperatures could limit their survival and, consequently, their effectiveness in controlling host populations. Fertility is a crucial factor for the growth and sustainability of insect populations and serves as an important indicator of how well natural enemy insects can control pest populations. Insects generally have a narrower temperature range for reproduction, making their fecundity particularly sensitive to temperature changes [46]. The same study found that short periods of low temperature can impair the reproductive capacity of *T. drosophilae*. Key indicators for predicting insect population dynamics include the net reproductive rate (*R*_0_), intrinsic rate of increase (*r*_m_), finite rate of increase (*λ*), and mean generation period (*T*). These metrics illustrate the potential for population growth under different environmental conditions [47], with the intrinsic rate of increase (*r*_m_) directly correlating with population trends [48, 49].

After a 12-h exposure to low temperatures, male *T. drosophilae* showed increased mating activity between 15:00 and 17:00. However, as the treatment temperature decreased, mating frequency dropped, and the duration of pupal beating increased. Additionally, the low-temperature treatment led to a shorter development time and reduced lifespan for adult *T. drosophilae*, as well as lower age-specific fecundity. On the other hand, both high and low temperatures during brief treatment periods negatively impacted the growth, development, and overall population increase of *T. drosophilae*, ultimately reducing their potential for biocontrol.

## Conclusions

Short-term cold temperatures impaired the reproductive ability of *T. drosophilae*, hindering their growth and reducing their effectiveness as biocontrol agents.

## Data Availability

No datasets were generated or analyzed during the current study.
